# Future-Frame Prediction for Fast-Moving Objects with Motion Blur

**DOI:** 10.3390/s20164394

**Published:** 2020-08-06

**Authors:** Dohae Lee, Young Jin Oh, In-Kwon Lee

**Affiliations:** Department of Computer Science, Yonsei University, Seoul 03722, Korea; dlehgo1414@yonsei.ac.kr (D.L.); skrcjstk@yonsei.ac.kr (Y.J.O.)

**Keywords:** machine physical reasoning, future frame prediction, motion blur

## Abstract

We propose a deep neural network model that recognizes the position and velocity of a fast-moving object in a video sequence and predicts the object’s future motion. When filming a fast-moving subject using a regular camera rather than a super-high-speed camera, there is often severe motion blur, making it difficult to recognize the exact location and speed of the object in the video. Additionally, because the fast moving object usually moves rapidly out of the camera’s field of view, the number of captured frames used as input for future-motion predictions should be minimized. Our model can capture a short video sequence of two frames with a high-speed moving object as input, use motion blur as additional information to recognize the position and velocity of the object, and predict the video frame containing the future motion of the object. Experiments show that our model has significantly better performance than existing future-frame prediction models in determining the future position and velocity of an object in two physical scenarios where a fast-moving two-dimensional object appears.

## 1. Introduction

Through visual observation, humans can perform quick and accurate physical reasoning. Many decisions in everyday life are made based on this physical reasoning ability. For example, if we play Jenga, we can infer the stable state of the stacked blocks to determine which block to extract without toppling the tower. Additionally, if we recognize that a threatening object is rapidly flying towards us, we can move our bodies quickly to avoid collision. For a machine to make similar reasonable judgments and actions to solve problems alongside humans, a considerably fast and accurate physical reasoning capability is required. Hence, research is necessary to help machines make reasonable physical inferences.

To meet this requirement, several studies have been conducted in which a machine analyzes the physical properties of an object in an image and a video sequence using deep-learning-based methods. For example, studies have been conducted to infer the stability of stacked blocks from images [1,2,3] or to infer physical properties, such as mass or density, of objects from video sequences, where collisions occur between the objects [4,5]. Studies have also been conducted to predict future movements of objects [6,7] or predict physical properties and their changes [5,8,9] in a short video sequence. However, in most of these studies, only videos of clearly captured objects have been focused upon. Although some studies have been conducted using video data with a small motion blur added to assume realistic video data [5,10], experiments were not performed with video data where a strong motion blur was captured.

Motion blur occurs when a fast-moving object is captured using a regular camera. We can use an ultra-high-speed camera to film the object clearly; however, there are several restrictions, because a significant amount of power, large storage capacity, and significant computations are required for processing. Therefore, it is necessary to perform physical reasoning on video data where motion blur occurs. However, this is challenging with existing methods that only handle normal video sequences. Many studies have been conducted to increase the accuracy on blurry images in various research fields [11,12]; this increase is also required in the field of physical reasoning. Additionally, the number of frames that can be used for physical reasoning may be limited, because fast-moving objects deviate relatively quickly from the camera range. Thus, physical reasoning should be performed with as few frames as possible.

We, therefore, propose a model consisting of three stages (recognition, prediction, and reconstruction) to predict an object’s future movement from a short video sequence where motion-blurred fast-moving objects are observed. The recognition model is named motion encoder, and it is a network that recognizes the states of an object, i.e., position and velocity, in the input frames and the shutter speed of the camera. Because the object moves fast, it is essential to perform physical reasoning with as few frames as possible, and our recognition model can recognize the object’s accurate motion information with only two input frames. The prediction network model is a network that receives the object states recognized by the motion encoder and predicts the future object states. Finally, the motion decoder model reconstructs the video frames using the predicted object states and the shutter speed. By generating a synthetically simulated motion-blurred video dataset and using it as training data, it is possible to train our model to accurately recognize, predict, and reconstruct an object’s movement in a motion-blurred video.

To verify the performance of the proposed method, we generated synthetic simulation datasets for two physical scenarios in which the object moves fast and collides with the walls. Consequently, our model accurately recognized the object states and shutter speed in the motion-blurred video and continuously predicted the subsequent future object states and frames from the two initial input frames that were almost similar to the simulation results. We showed that accurate physical reasoning is also possible for real videos by performing unsupervised fine-tuning using a small amount of real video data.

## 2. Related Work

Humans do not need to calculate Newtonian physical equations to make physical inferences in real life. Instead, we create approximate predictions using intuitive inferences that are instinctively obtained and reinforced through lifetime experience. This is called “intuitive physics” in the field of cognitive science. Intuitive physics is at the core of artificial intelligence and has been actively researched in recent years [13]. Meanwhile, with the development of graphical processing units in recent years, deep neural network-based methods have shown excellent results in various fields [14,15,16], and physical inferences using deep neural network have been attempted in several studies.

### 2.1. Learning Implicit Physics

In one of the first studies [1] in which the concept of intuitive physics was incorporated into the field of machine physical reasoning, a model was proposed to perform simulation-based stochastic physical reasoning instead of deterministic physical reasoning. The proposed model [1] could determine whether stacked blocks were stable by receiving the state of the blocks as input. Subsequently, studies were conducted to determine the stability of boxes from images using deep learning-based models [2,3] and predict the movement of objects when a specific external force was applied [7,10]. Movements based on external forces were predicted not only under limited conditions, such as with stacked boxes or billiards, but also in general situations of daily life with various objects [6]. For example, it was possible to predict the future movement of a cup or a chair when a specific external force was applied. Meanwhile, studies were conducted to determine whether a robot could arrange objects in real life based on objects in an image [17]. Other studies were conducted to allow agents to interact directly with physical objects in real life to learn hidden intuitive physics [18,19]. There have also been studies to implicitly infer the dynamics of each object from the video of multiple moving objects and predict future frames [20,21]. Based on the human ability to identify physical causality through visual observations, a new problem called “counterfactual learning of objects mechanics” was presented [22]. The model proposed in that study predicted how the movement of an object might have changed when the initial state of the object changed slightly after a single observation of the physical movement of the object. In all the studies described in this section, the object states or physical variables were not explicitly or directly deduced. Rather, problems were presented that could be solved only when physical reasoning was possible, and methods were proposed to solve them.

### 2.2. Learning Explicit Physics

Meanwhile, studies have been conducted to explicitly deduce object states or physical variables. Models for inferring physical variables, such as mass and density, after observing a video of moving objects have been proposed [23,24,25]. Methods have also been proposed to infer the force applied to objects or the objects’ current speed from images [26,27]. Some studies were conducted to predict future object states based on the current object states [28], making it possible to recognize the current state of an object from visual input and predicting subsequent frames [29]. There was also a model that expressed the representation of the dynamics and states of objects separately in the latent space from a video and performed future predictions based on it [30]. Another model defined rigid dynamics as a linear complementary problem and found its variables using an auto-encoder to predict future movements of the object from video input [31]. Another method was proposed to obtain the property vector for each object from the video to predict the future trajectories of objects and infer the interpretable physical values, such as mass or rebound coefficients, using principle component analysis [4]. Some studies were conducted to predict the future movement of balls rolling in elliptical bowls [8,32], and others were conducted to predict future object states and future frames by inputting short video sequences [9,33] using the spatial transform network [34]. A study was also conducted to predict the future trajectories of a bouncing ball [5] by using a variational recurrent neural network [35].

### 2.3. Learning for Real Scenes

In some studies, only synthetic data generated from physical simulations were used to perform physical reasoning, whereas, real data were used in others. For example, motion parameters, including positions and poses, for fast moving objects were estimated in a study [36]. A method was proposed to infer the collision parameters of objects and reconstruct the 3D scene, including collisions, after watching a video in which the collision between two objects was obscured by a curtain [37]. These methods can be applied to real scenes without a large number of data, because they do not use machine-learning techniques. However, there are many studies in which machine learning-based methods required a large amount of real data. For example, studies were conducted to predict the future movement of objects when an external force was applied [6] or to predict future frames by viewing a video of robot arms on a table swinging various objects [38]. In another study, the future movement of objects in a video was predicted using physical expression as a prior [39]. In several studies, real data were used to train the models [8,32]. However, generation of large amounts of real data is costly and results in limited utility. As an alternative, attempts have been made in several studies to handle real data with models trained on synthetic data. Some of these models were re-trained using real data to apply to real images [10]. Some other models were trained with synthetic data and applied directly to real data by generating synthetic data according to domain randomization techniques [5,40].

All the existing methods of processing real data introduced so far were designed to process only images or videos of clearly captured objects. Some of them added weak motion blur to the data [5,10], but did not deal with the data where severe motion blur was captured. Our proposed method can recognize the state of objects in the synthetic and real video sequences where the motion blur is severely captured. Additionally, since it is expensive to collect labeled real data, we propose a new method to process real data using a small number of unlabeled real data. This is possible by training a model with synthetic data and then fine-tuning the model using a small amount of real data in an unsupervised manner.

## 3. Methods

### 3.1. Synthetic Data Generation

**Figure 1:**
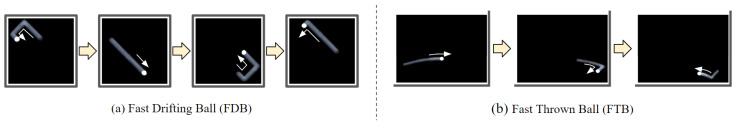
Two physical scenarios used to generate the dataset.

We aim to recognize and predict the states of a fast-moving object for both synthetic and real video data. The model for synthetic data can be trained using synthetic data, but it is desirable to use real data to train a model that processes real data [5,10,40]. However, it is not easy to use real data for model training, because it is complicated to generate a large amount of real data under various camera conditions with different initial positions and velocities of an object. As an alternative, we simulated the motion of an object using a physics engine to generate synthetic data and then used it to train the model. In this study, we generated synthetic data by simulating two physical scenarios: a fast-drifting ball (FDB) and fast-thrown ball (FTB). An FDB drifts rapidly with a fully elastic collision with the walls on four sides in a zero-gravity environment. An FTB is quickly thrown into a wall on the right side in a gravitational environment. In both scenarios, we generated datasets for model training by setting various initial object locations, velocities, and camera conditions, running multiple simulations, and rendering simulation results (Figure 1).

**Figure 2:**
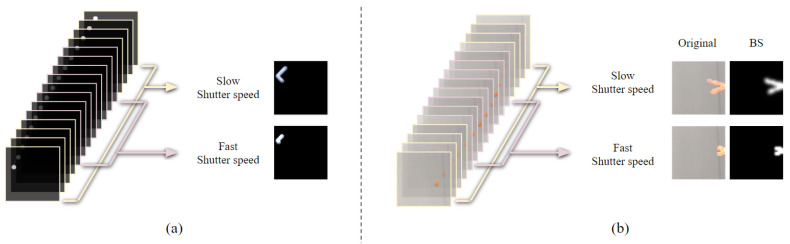
Synthesizing one blurry frame using consecutive clear frames, assuming different shutter speeds. (**a**) Result of synthesizing a blurry frame using synthetic frames. (**b**) Result of synthesizing a blurry frame using frames captured using a high-speed camera. BS represents the background subtraction from the synthesized original blurry frame.

Unlike filming a fast-moving object using a regular camera, the synthetic frames generated by rendering the simulation results were clear without motion blur. Therefore, it was necessary to convert clear synthetic frames into motion-blurred frames. We synthesized one blurry frame from 16 consecutive sharp frames by applying the method of synthesizing a blurry image with consecutive sharp frames captured using an ultra-high-speed camera for use as a dataset of a network model for image deblurring [41]. Additionally, we adjusted the number of sharp frames used for synthesizing the blur frame to reflect the phenomenon that the motion-blur intensity varies depending on the camera shutter speed. For example, we used 16 synthetic frames for generating the blur frame with slow shutter speed, but we used fewer synthetic frames for generating a blur frame with a faster shutter speed. Figure 2a shows examples of synthesizing a blurry frame with synthetic frames, and Figure 2b shows the process of synthesizing a blurry frame with real video frames captured using an ultra-high-speed camera. As observed from the figure, a blurry frame was synthesized using all 16 sharp frames as a result of assuming a slow shutter speed, and eight middle-sharp frames were used as a result of assuming a fast shutter speed. These synthesized blurry frames with position, velocity, and shutter-speed labels of the object were used as the dataset for training.

### 3.2. Future-Frame Prediction Network

**Figure 3:**
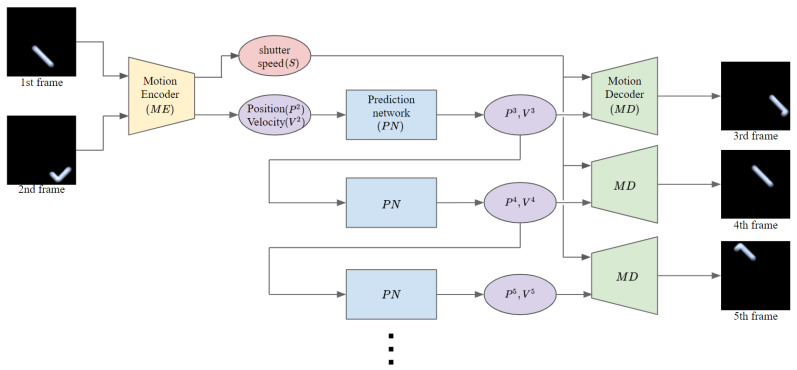
Overview of the proposed model.

The future-frame prediction network proposed to predict the next movement of a motion-blurred fast-moving object consists of three components: motion encoder (*ME*), prediction network (*PN*), and motion decoder (*MD*). The motion encoder recognizes the shutter speed and the object state (i.e., the position and velocity of the object) from two blurry input frames. The prediction network predicts the object state in the next frame from the current object state. The motion decoder generates the future blurry frame using the predicted object state and the recognized shutter speed. By performing recursive inferences that input the predicted object state, which is the output of prediction network, back into prediction network, we can generate subsequent future blurry frames from the first two input blurry frames (Figure 3).

**Figure 4:**
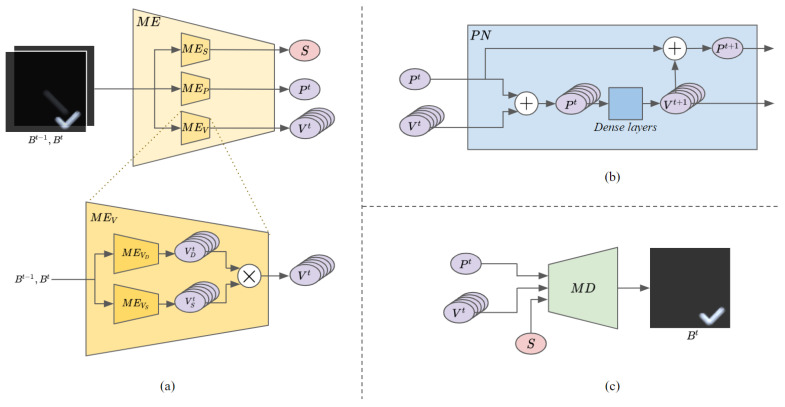
Overview of model details: (**a**) motion encoder; (**b**) prediction network; (**c**) motion decoder.

Figure 4 shows the detailed structures of each network constituting the model. Let $B^{t-1}$ and $B^{t}$ be two input blurry frames of the model. Motion encoder (Figure 4a), comprising a position encoder ($ME_{P}$), velocity encoder ($ME_{V}$), and shutter-speed encoder ($ME_{S}$), recognizes the shutter speed (*S*), velocity ($V^{t}$), and position ($P^{t}$) of the object in the second input frame, $B^{t}$. The velocity information recognized by the velocity encoder includes the consecutive velocities of the object in all 16 sharp frames used to generate the blurry frame. The position information recognized by the position encoder is the position of the object in the last sharp frame, i.e., the 16th frame. Our motion encoder receives two consecutive blurry frames as input to recognize the object states accurately, because it is difficult to specify the direction of the object’s movement using only a single blurry frame. Moreover, because the intensity of the motion blur may be generated differently, even at the same object velocity, depending on the shutter speed of the camera, object state cannot be accurately recognized using only one input frame. The velocity encoder consists of a direction encoder ($ME_{V_{D}}$) and speed encoder ($ME_{V_{S}}$) that recognize the direction ($V_{D}$) and speed ($V_{S}$), respectively, and it outputs the final velocity value by multiplying the recognized direction and speed. The recognized direction is the direction of movement of the object along each axis, and the recognized speed is the magnitude of the velocity of the object along each axis. For example, for any object moving in the positive direction of the x-axis, the direction in the x-axis is a constant positive value which is 1. Conversely, for an object moving in the negative direction of the x-axis, the direction of the x-axis is a constant negative value −1. If an object changes its travel angle slightly, but the direction for each axis is the same, there is no change in the direction value, but the speed value for each axis changes. Prediction network (Figure 4b) predicts the object velocity of the next frame ($V^{t+1}$) by inputting the continuous position information, which is calculated from the recognized velocity ($V^{t}$) and position ($P^{t}$), into a neural network comprising dense layers. The object position of the next frame ($P^{t+1}$) is then calculated using the input position ($P^{t}$) and the predicted velocity of the next frame ($V^{t+1}$). Motion decoder (Figure 4c) is a network that reconstructs the blurry future frame from the states of the object and shutter speed. It can generate future frames based on the object states predicted by the prediction network and the shutter speed recognized by the motion encoder.

### 3.3. Training Scheme

In this section, we describe the training scheme for the proposed network model. Because it is challenging to collect sufficient real data to train a deep neural network model, we generated synthetic data using realistic physical simulation for training the model. After training the network using this synthetic data, a fine-tuning step was performed using a small number of real data to apply the model to real video data.

#### 3.3.1. Training with Synthetic Data

Because the synthetic data contains the object state labels, training with synthetic data was conducted in a supervised manner. First, the motion encoder loss, $L_{ME}$, is defined as follows:

(1) $$L_{ME}=L_{p}+L_{vd}+L_{vs}+L_{s},$$

where the position loss, $L_{p}$, velocity-direction loss, $L_{vd}$, velocity-speed loss, $L_{vs}$, and shutter-speed loss, $L_{s}$, are given as follows:

(2) $$L_{p}=MSE\left(P^{t},{\hat{P}}^{t}\right),\;L_{vd}=CE\left(V_{D}^{t},{\hat{V}}_{D}^{t}\right),\;L_{vs}=MSE\left(V_{S}^{t},{\hat{V}}_{S}^{t}\right),\;L_{s}=MSE\left(S,\hat{S}\right),$$

where $P^{t}$, $V_{D}^{t}$, $V_{S}^{t}$, and *S* are the ground-truth labels of position, direction, speed, and shutter speed, respectively, and ${\hat{P}}^{t}$, ${\hat{V}}_{D}^{t}$, ${\hat{V}}_{S}^{t}$, and $\hat{S}$ are corresponding values recognized by the motion encoder. MSE and CE represents the mean squared error and cross entropy error, respectively. Note that the velocity direction error is measured by the cross entropy error, because the velocity direction is one of the positive or negative directions along the each axis.

The prediction network loss, $L_{PN}$, is defined as the mean squared error between the ground-truth velocity, $V^{t+1}$, and the predicted object velocity in the next frame, ${\hat{V}}^{t+1}=PN\left(P^{t},V^{t}\right)$, as follows:

(3) $$L_{PN}=MSE\left(V^{t+1},{\hat{V}}^{t+1}\right),$$

The position value is not reflected in the loss term of the prediction network because the next position can be calculated by a combination of the previous position and the velocity: $P_{t+\Delta t}=P_{t}+\Delta t\times V_{t}$.

The motion decoder loss, $L_{MD}$, is the mean squared error between the ground-truth blurry frame, $B^{t+1}$, and the reconstructed frame, ${\hat{B}}^{t+1}=MD\left(P^{t+1},V^{t+1},S\right)$, generated based on the shutter speed, *S*, next object position, $P^{t+1}$, and velocity, $V^{t+1}$, as follows:

(4) $$L_{MD}=MSE\left(B^{t+1},{\hat{B}}^{t+1}\right),$$

During the training phase using synthetic data, the model is trained together by specifying the following loss as an objective function that includes all the losses defined for each sub-network:

(5) $$L_{Synth}=\lambda_{ME}L_{ME}+\lambda_{PN}L_{PN}+\lambda_{MD}L_{MD},$$

where the weights, $\lambda_{ME}$, $\lambda_{PN}$, and $\lambda_{MD}$, used for scaling each network-loss function, were experimentally determined to be 10, 100, and 100, respectively.

#### 3.3.2. Fine-Tuning with Real Data

We performed the fine-tuning process using real data to enable the model trained with synthetic data to more accurately predict the movement of objects in the real data. The model was trained using synthetic data in a supervised manner, because there were large amounts of object-state labels in the synthetic data. However, there was no object-state label for the real data. Thus, fine-tuning was performed in an unsupervised manner. We performed fine-tuning of the motion encoder and prediction network one-by-one such that the model would recognize and predict the exact object states from the real data. All real video data were used after the background subtraction during the pre-processing stage to highlight only the motion information of the object in the video. We used K-nearest neighbours-based background subtraction algorithm [42] for the background subtraction.

The loss for fine-tuning the motion encoder using real data, $L_{EF}$, is given as follows:

(6) $$L_{EF}=MSE\left(B^{t},{\hat{B}}^{t}\right),$$

where $B^{t}$ is the ground-truth frame, and ${\hat{B}}^{t}=MD\left(ME\left(B^{t-1},B^{t}\right)\right)$, which is reconstructed by directly inputting the object state recognized by the motion encoder into the motion decoder. For fine-tuning the motion encoder, all weights of the prediction network, motion decoder, and the weights constituting the convolution neural network parts of the motion encoder were frozen, and only the weights of the last few fully connected layers of the motion encoder were updated via back propagation.

The prediction network fine-tuning loss, $L_{PF}$, used for fine-tuning the prediction network is as follows:

(7) $$L_{PF}=L_{NP}+L_{NR},$$

where the next-prediction loss, $L_{NP}$, and the next-reconstruction loss, $L_{NR}$, constituting the prediction network fine-tuning loss, are as follows:

(8) $$L_{NP}=MSE\left(V_{ME}^{t+1},V_{PN}^{t+1}\right)),\;L_{NR}=MSE\left(B^{t+1},{\hat{B}}^{t+1}\right)).$$

Thus, the next prediction loss is an error between the object velocity in the next frame, $V_{ME}^{t+1}=ME\left(B^{t},B^{t+1}\right)$, recognized by the motion encoder and the object velocity in the next frame, $V_{PN}^{t+1}=PN\left({\hat{P}}^{t},{\hat{V}}^{t}\right)$, predicted by the prediction network based on the previous object states recognized by the motion encoder. Next reconstruction loss is an error between a ground-truth frame, $B^{t+1}$, and a frame reconstructed based on object states predicted by the prediction network, ${\hat{B}}^{t+1}=MD\left(PN\left({\hat{P}}^{t},{\hat{V}}^{t}\right),\hat{S}\right)$. During prediction network fine-tuning, the weights of motion encoder and motion decoder were frozen, and only the prediction network was updated via back propagation.

### 3.4. Implementation Details

**Figure 5:**
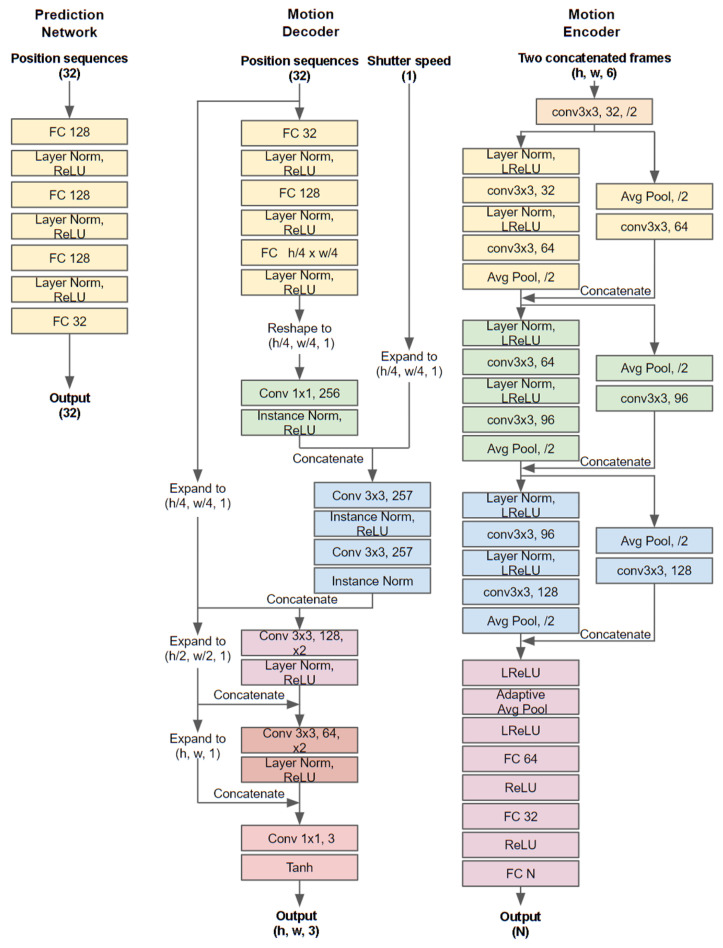
Detailed network architecture of the proposed model.

Our model was developed based on the source code provided in past research to remove blur effects by separating the blur information and content information from the blurred image [43]. The velocity-direction encoder, velocity-speed encoder, position encoder, and shutter-speed encoder in the motion encoder were from the same structure, and only the dimensions of the output differed from each other: 32 for the velocity encoder (2D coordinates per 16-time steps), two for the position encoder (2D coordinates per one-time step), and one for shutter speed. They all concatenated two input frames before processing and consisted of four stride convolution layers and three fully connected layers. The motion encoder had a symmetrical structure and consisted of three fully connected layers, 1 × 1 convolution layer, three residual blocks, and three transposed convolution layers. The prediction network consisted of four dense layers. The detailed architecture of the proposed model is shown in Figure 5. We used the Adam optimizer [44] in all the experiments and initialized the weights to follow a Gaussian distribution. The learning rate was initially set to 0.0002, and after 40 epochs, it decreased to 0 when it reached 2000 epochs. All the experiments were performed using a machine equipped with an i5-7500 core with two GTX 1080ti GPUs. With the FDB dataset, we trained our model for 700 epochs in batch sizes of 32. We trained our model for 140 epochs with an FTB-synthetic dataset in batch sizes of 32 and trained for three epochs more for motion encoder fine-tuning and five epochs for prediction network fine-tuning with FTB-real dataset in batch sizes of 16.

### 3.5. Extension to Multiple Objects

**Figure 6:**
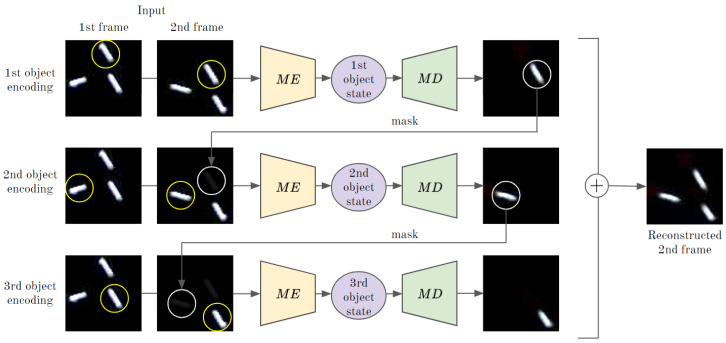
Encoding states of three objects.

Thus far, we had assumed that there was only one fast-moving object. However, multiple objects can be processed by repeatedly applying the motion encoder and motion decoder without modifying the model. If an input video has multiple moving objects, the motion encoder recognizes the state of the object located at the top in the frame, and the motion decoder reconstructs the frame of that object based on the recognized information. Subsequently, the reconstructed frame is used as a mask for the second input frame to erase the first recognized object. The frame where the first object is erased is input to the motion encoder to recognize the state of the second object. By repeating the recognizing, reconstructing, and masking process for as many times as the number of objects, all states for multiple objects can be recognized. Based on these recognized object states, the next movement of objects can be predicted using prediction network and the future frames can be constructed using the motion decoder (Figure 6).

If there are *n* moving objects, the synthetic loss, $L_{Synth}$, is defined as the sum of the motion encoder loss, prediction network loss for each object, and motion decoder loss as follows:

(9) $$L_{Synth}=\sum_{i=1}^{n}\left(\lambda_{ME}L_{ME}^{i}+\lambda_{PN}L_{PN}^{i}\right)+\lambda_{MD}L_{MD},$$

where $\lambda_{ME}$, $\lambda_{PN}$, and $\lambda_{MD}$ were experimentally determined to be 10, 100, and 100, respectively.

## 4. Results

**Figure 7:**
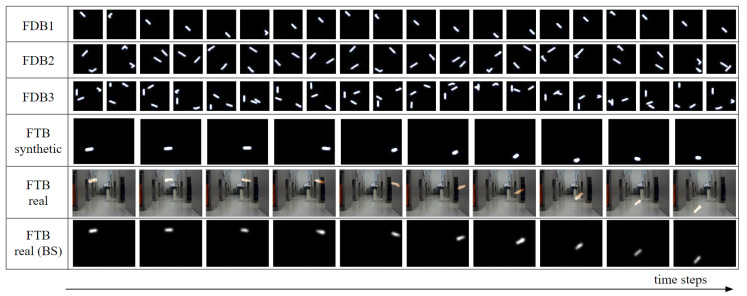
Dataset examples.

To demonstrate the performance of the proposed method, we conducted experiments on datasets of two different physical scenarios: FDB and FTB. As can be seen in Table 1 and Figure 7, FDB comprised only synthetic data, and FTB comprised synthetic and real data. We generated synthetic data using the Unreal Engine 4, and we generated real data using a Sony rx100 camera at 24 fps with a shutter speed of 1/25. The FDB dataset was divided into FDB1 to FDB3 according to the number of objects, and the FTB dataset was divided into FTB-synthesis and FTB-real, which comprised synthetic and real data, respectively. FDB1 and FDB2 were composed of 1000 video sequences, and FDB3 was composed of 2800 video sequences. FTB-synthetic was composed of 4800 video sequences. 100 of them were used for testing. FTB-real was composed of 94 video sequences, 30 of which were used for testing. Each video sequence in FDB dataset was composed of 20 frames of 64 × 64 frames, and each video sequence in FTB dataset was composed of 10 frames of 160 × 120 frames. Synthetic datasets had labels for object states and shutter speed, whereas real data had no labels. Examples of FDB and FTB datasets can be seen in Figure 7. It shows the frames that make up the video sequence of each dataset sequentially along the time-step. FTB Real (BS) means the background subtraction result of FTB-real.

### 4.1. FDB

**Figure 8:**
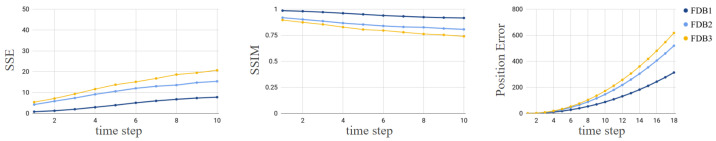
Errors for each prediction time step from FDB1 to FDB3.

**Figure 9:**
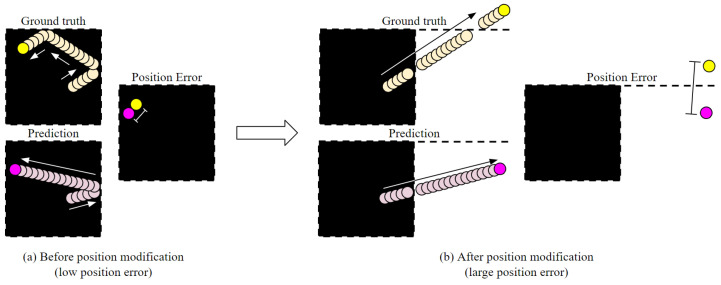
Position conversion process for position error calculation in fast-drifting ball (FDB).

**Figure 10:**
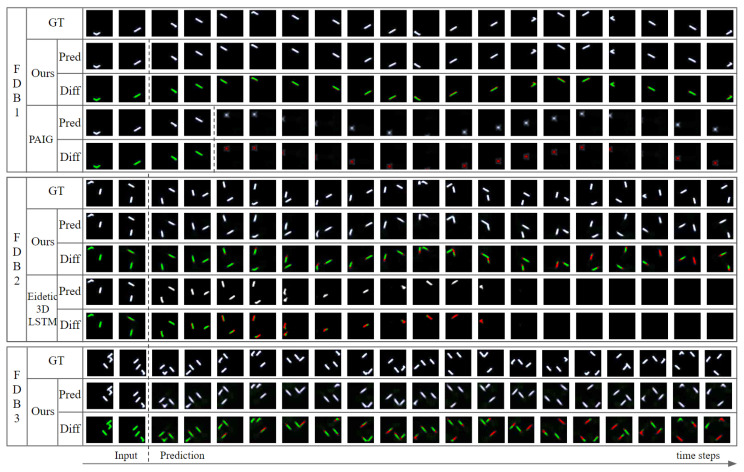
Ground-truth (GT) and results predicted by our model (Ours) and comparative models (PAIG, Eidetic 3D LSTM) from FDB1 to FDB3. Pred represents the frames predicted by the model, and Diff represents the difference between GT and Pred. The green area represents the correct part, and the red area represents the wrong part of the model’s prediction. PAIG receives four initial frames, and our model and Eidetic 3D LSTM receive two.

The proposed model successfully recognized the object state and predicted the future object states and frames from a motion-blurred video in experiments using the FDB dataset. We performed the experiments to predict the next 18 frames and future object states when only the first two frames were given among 20 frame sequences constituting FDB data. Table 2 and Figure 8 shows the sum of squared error (SSE) and the structural similarity (SSIM) between the predicted future frames and ground-truth frames in the experiments using FDB1, FDB2, and FDB3. It also shows the position error (PE) between the predicted object position and ground-truth position. For calculating the PE, just comparing the difference between the naive positions cannot be a valid performance indicator, because the objects in the FDB elastically collide with the walls in all directions and continuously drift through a limited space. Therefore, we compared the differences between the deformed positions: whenever the object collides with a wall and the velocity changes, the velocity is symmetrically transformed based on the collided wall to calculate a new position. In other words, the PE is calculated after correcting the position as if the object is going through the wall and moving in one direction (Figure 9). As shown in Table 2, the error increases as the number of objects in the scene increases. In FDB1 with one object, SSE and PE are the lowest, SSIM is the highest. As the number of objects increases, SSE and PE become larger and SSIM becomes smaller. This shows that the accuracy of motion prediction decreases as the number of objects increases. The graph in Figure 8 shows the errors at each prediction time-step. As can be observed in the graph, the error tends to increase as the prediction time-step increases. For SSE and PE, the error increases as the prediction time-step increases, and SSIM decreases as the prediction time-step increases. This means that the accuracy of motion prediction decreases as the predicted time-step increases. This is because the errors accumulated via the iterative process of reusing the predicted object states as an input of the prediction network. However, as can be seen in Figure 10 in the “Ours’’ row, our model predicted the intricate movements of the objects reasonably, even when the number of objects and predicted time-step increased. If there was one object in the scene, our model predicted future frames similar to the ground-truth up to the last 18th frame. If two or more objects were in the scene, although the error was larger than the single-object case, our model still made reasonable predictions.

Because there has been no prior study about predicting future frames and movements of an object on a video where intense motion blur occurs because of the fast movement of the object, we compared our performance with those in recent studies for predicting the future frames of a video. We trained a PAIG model [9], which predicts the future object states and frames for a sharp video, and the Eidetic 3D LSTM model [45], which predicts only the frames without predicting the future object states, using the FDB dataset. We then compared their tested results with those generated by our model. First, we trained the PAIG model to predict future frames by receiving four blurred frames of FDB1, because PAIG requires at least four input frames to predict future motion. We compared the results of PAIG with those of our model under three metrics: PE, SSE, and SSIM. We also compared the time it takes to predict a frame: Execution time. As can be seen in the top row of Table 3, although PAIG received more frames than our model, our results were better in all three metrics. Our model showed 33 times lower PE than the comparative model. Additionally, our model had a lower SSE and a higher SSIM than the comparative model, which means that our model predicted more accurate frames. The PAIG model could not correctly recognize and predict the object states nor could it reconstruct the frame if there was motion blur on the object. Figure 10 shows the frames predicted by our model and the compared models. Our model predicted correctly until the last time-step, while the comparative model continued to fail to predict correctly. The execution time was longer than that of the PAIG model, but the processing time per frame is about 0.012 s, and real-time processing is possible for videos of about 80 fps. Also, since our model has not been optimized to reduce execution speed, there is room for further improvement in speed. The Eidetic 3D LSTM model was trained using FDB2, and the results were measured using the previous two metrics (i.e., SSE and SSIM) and the execution time. We omitted PE, because Eidetic 3D LSTM model does not immediately predict object states. As shown at the bottom of Table 3, our model performed better in both metrics. As can be seen in Figure 10 “FDB2’’ row, our model made reasonable predictions similar to the ground-truth, although the error increased as the predicted time step increased. However, the Eidetic 3D LSTM made relatively reasonable predictions at first, but errors rapidly increased as the prediction time step increased. Moreover, after several time-steps, errors such as objects disappearing or objects not being drawn occurred. For FDB2, the execution time of our model was longer than that of the comparative model, but the processing time per frame is about 0.023 s, and real-time processing is possible for videos of about 40 fps.

**Figure 11:**
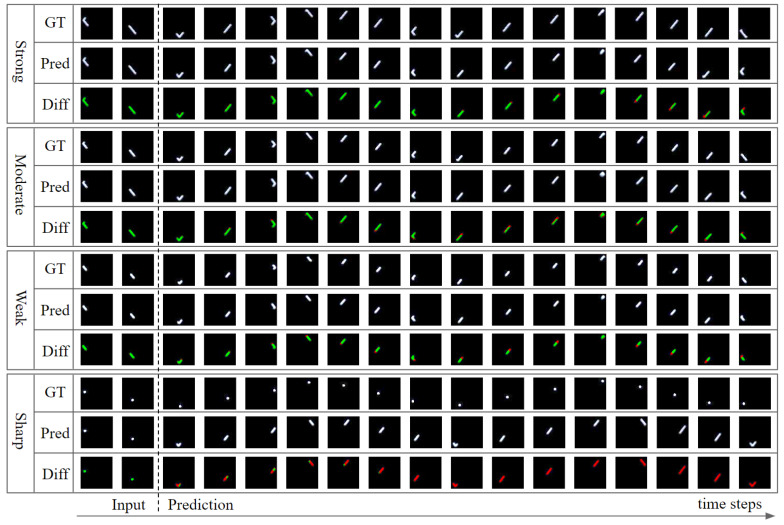
Result of future frame prediction for a video sequence with the same object states but different shutter speeds. GT is the ground-truth, Pred is the model’s prediction, and Diff is the difference between Gt and Pred. The green area represents the correct part, and the red area represents the wrong part of the model’s prediction.

Even if we film the same scene using the same camera at the same frame rate, various motion blur can occur, depending on the shutter speed of the camera. Thus, the model trained with the training dataset consisting only of videos of certain shutter speeds is difficult to apply to videos having other shutter speeds. Therefore, we organized FDB into videos assuming various shutter speeds such that our model could be applied well, regardless of the shutter speeds. To compare the accuracy of our model according to the degree of motion blur, we constructed an FDB1 test set having four different shutter speeds and compared the errors: videos with strong, moderate, weak motion blur, and no motion blur (sharp). Consequently, our model made reasonable predictions from strong motion blur to weak motion blur, but not in sharp video (Figure 11). As can be seen in Table 4, our model obtained the highest accuracy when the motion blur was weak, and the accuracy decreased as the motion blur became stronger. The accuracy was lowest when there was no motion blur at all. This is because, when there was a moderate motion blur, our model could obtain additional information regarding the object states from it. However, the stronger the motion blur, the more difficult it was to recognize the object states. If no motion blur occurs, owing to the considerably short shutter speed, the error increases, because additional information about the object states cannot be obtained from the motion blur.

The proposed velocity encoder was divided into a direction encoder and a speed encoder. The final velocity was recognized by multiplying the direction and speed measured by each sub-network. By configuring the velocity encoder in this manner, it performed better than when the velocity encoder directly recognized the velocity. To verify this, we experimented with FDB1 and compared the velocity error and the position error. Velocity error is the mean-squared error between the ground-truth velocity and the predicted velocity and the position error is the mean-squared error between the ground-truth position and the predicted position. As shown in Table 5, the velocity encoder constructed using the proposed method performed much better in both metrics. This is because it could be better trained by dividing and solving the velocity recognition problem into two easier sub-problems.

### 4.2. FTB

**Figure 12:**
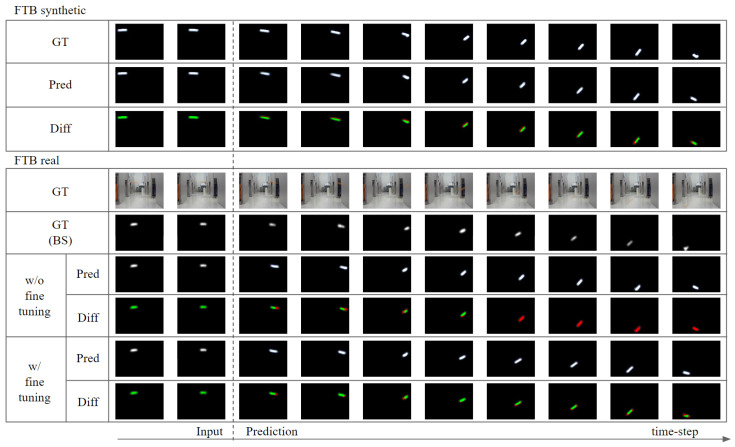
Future-frame prediction result after receiving two initial frames for FTB. (**top**) The model predicted results (PRED) and ground-truth (GT) for FTB-synthetic. (**bottom**) Results of the model trained only with FTB-synthetic on FTB-real (w/o fine-tuning), results after fine-tuning with FTB-real (w/ fine-tuning), ground-truth with background subtracted (BS), and ground-truth. In Diff, the green area represents the correct part, and the red area represents the wrong part of the model’s prediction.

We conducted experiments with FTB to show that our model works well for real data. Because the FTB-real data do not have labels for object states, we used SSE and SSIM between the ground-truth frames and predicted frames as a comparative metric for quantitative comparison of the results. When we trained our model using FTB-synthetic data, it could predict the future frames of FTB synthetic accurately (Table 6 top, Figure 12 top), but not for FTB real (Table 6 bottom 1st row, Figure 12 bottom “w/o fine tuning” row). However, by fine-tuning the model, which was pre-trained using FTB-synthesis, using FTB-real, it was able to predict future frames accurately in FTB-real. As can be seen in Table 6 bottom, fine-tuning the prediction network using only the next-reconstruction loss ($L_{NR}$) reduced accuracy of the model, while fine-tuning the prediction network using only the next-prediction loss ($L_{NP}$) increased accuracy. The accuracy was higher if prediction network was fine-tuned using both reconstruction loss and prediction loss ($L_{PF}$). The highest accuracy was obtained when the fine-tuning was performed for both the motion encoder and the prediction network (ALL). An example of the experimental results for the fine-tuning is shown in Figure 12.

## 5. Conclusions

In this paper, we proposed a method for recognizing the position and velocity of an object from a video sequence containing motion blur caused by the object’s fast movement and predicting future video frames containing the future motion of the object. The proposed model continuously predicted the subsequent state of the object by inputting only the first two frames of the video; this was accomplished by obtaining additional information regarding the motion of the object from motion blur. In addition, by dividing the velocity into direction and speed, more accurate velocity information can be recognized. This model can not only manipulate video data at various shutter speeds, but also predict the state of multiple objects appearing in the video. Our model successfully recognized and predicted the object state of the actual video data as well as the synthetic data by applying unsupervised fine-tuning of the pre-trained model with synthetic data using a small number of real data. To the best of our knowledge, this is the first attempt to recognize and predict the state of an object in a video sequence where strong motion blur occurred due to the rapid movement of the object. We have shown that the proposed model is remarkably superior to the existing methods in predicting the future movement of fast-moving objects. This allows the machine to accurately identify the position and velocity of fast-moving objects using a regular camera.

The proposed method successfully recognized and predicted the fast-moving objects’ movements, but there is still room for improvement. First, we focused on accurately performing physical reasoning using as few frames as possible, but did not perform a model optimization process to maximize execution speed. Therefore, the inference speed can be improved by searching for an optimal model in consideration of performance and speed. Additionally, this study was only focused on 2D moving objects and prediction of future motion of fast-moving objects in 3D space was not intended. We also considered only the translational position and velocity as the state of the object in this study. In the future, we may extend the results of this study to consider more properties, such as angular velocity and various object shapes, to predict future motions of moving objects in 3D space. In this study, we processed the real data after removing the background; however, it may be challenging to apply these techniques if the background is not static. Improvement of these shortcomings will also be an exciting future research topic. 

## Figures and Tables

**Table 1:** Dataset summary.

	FDB			FTB	
	FDB1	FDB2	FDB3	FTB Synthetic	FTB Real
generating method	synthetic	synthetic	synthetic	synthetic	real
number of objects	1	2	3	1	1
train dataset amount	900	900	2700	4700	64
test dataset amount	100	100	100	100	30
frame size	64 × 64	64 × 64	64 × 64	160 × 120	160 × 120
number of frames per video	20	20	20	10	10
object states/shutter speed labels	exists	exists	exists	exists	not exists

**Table 2:** Errors according to the number of objects in FDB.

	FDB1	FDB2	FDB3
SSE	4.40	10.61	13.81
SSIM	0.948	0.855	0.809
PE	107.52	178.86	211.44

**Table 3:** (top) Position error (PE), sum of squared error (SSE), and similarity index (SSIM) obtained by the PAIG model and our model for FDB1. (bottom) SSE and SSIM obtained by the Eidetic 3D LSTM model and our model for FDB2. Execution time represents the average time (seconds) to predict one frame.

Method	PE	SSE	SSIM	Execution Time
PAIG	3549.24	6.70	0.816	0.006
Ours	107.52	4.40	0.948	0.012
**Method**	**PE**	**SSE**	**SSIM**	**Execution Time**
Eidetic 3D LSTM	-	12.98	0.850	0.008
Ours	178.86	10.61	0.855	0.023

**Table 4:** Position error according to the degree of motion blur.

Motion Blur Degree	Strong	Moderate	Weak	Sharp
Position error	470.52	264.27	7.65	1338.03

**Table 5:** Position error and velocity error of the proposed velocity encoder and the one that directly recognized the velocity without dividing into direction and speed.

	Position Error	Velocity Error
w/ velocity encoder division	107.52	16.46
w/o velocity encoder division	3144.60	116.36

**Table 6:** Quantitative results of experiments on FTB. (top) Test results for FTB synthetic after training model with FTB synthetic. (bottom) Test results for FTB real depending on the fine-tuning method.

Model	SSE	SSIM
No fine tuning	10.75	0.980
**Model**	**SSE**	**SSIM**
No fine tuning	13.49	0.968
$L_{NR}$ only	14.82	0.963
$L_{NP}$ only	10.24	0.975
$L_{PF}$ only	9.30	0.976
All	9.15	0.977
